# Supplementary material for: The Dynamics of Cryptococcus neoformans Cell and Transcriptional Remodeling during Infection
Source: Cells. 2022 Dec 2;11(23):3896. doi: 10.3390/cells11233896 (PMC9740611; doi:10.3390/cells11233896)
Supplement: Supplementary file 1 [file cells-11-03896-s001.zip › cells-2015164-supplementary/Table S3 - SHIRPA.doc]

Table S17. SHIRPA (evaluated parameters)

| Functional categories | Parameters (Lackner et al. 2006; Pedroso et al. 2010; Santos et al., 2014; Costa et al., 2016). |
| --- | --- |
| Muscle tone and strength | Grip strength, body tone, limb tone, abdominal tone |
| Motor behavior | Body position, tremor, locomotor activity, pelvic elevation, gait, tail elevation, trunk curl, limb grasping, wire maneuver, negative geotaxis |
| Neuropsychiatric state | Spontaneous activity, transfer arousal, touch escape, positional passivity, biting, fear, irritability, aggression, vocals |
| Autonomous function | Respiration rate, defecation, urination, palpebral closure, piloerection, skin color, heart rate, lacrimation, salivation |
| Reflex and sensory function | Startle response, visual placing, pinna reflex, corneal reflex, toe pinch, righting reflex |
